# Syndecan-1 Promotes Hepatocyte-Like Differentiation of Hepatoma Cells Targeting Ets-1 and AP-1

**DOI:** 10.3390/biom10101356

**Published:** 2020-09-23

**Authors:** Péter Hollósi, Lóránd Váncza, Katalin Karászi, Katalin Dobos, Bálint Péterfia, Enikő Tátrai, Péter Tátrai, Tibor Szarvas, Sándor Paku, László Szilák, Ilona Kovalszky

**Affiliations:** 1 First Department of Pathology and Experimental Cancer Research, Semmelweis University, Üllői út 26, H-1085 Budapest, Hungary; phollosi@yahoo.com (P.H.); vancza_lorand@yahoo.com (L.V.); tika0604@gmail.com (K.K.); kdobos@gmail.com (K.D.); peterfiab@gmail.com (B.P.); tatraien@gmail.com (E.T.); soobaab@gmail.com (P.T.); sztibusz@gmail.com (T.S.); paku.sandor@med.semmelweis-univ.hu (S.P.); 2 Tumor Progression Research Group of Joint Research Organization of Hungarian Academy of Sciences and Semmelweis University, Széchenyi István tér 9, H-1051 Budapest, Hungary; 3 Faculty of Information Technology and Bionics, Pázmány Péter Catholic University, Práter u. 50/A, H-1083 Budapest, Hungary; 4 Department of Experimental Pharmacology, National Institute of Oncology, Ráth György utca 7-9, H-1122 Budapest, Hungary; 5 Solvo Biotechnology, Irinyi József utca 4-20, H-1117 Budapest, Hungary; 6 Department of Urology, Semmelweis University, Üllői út 78/B, H-1082 Budapest, Hungary; 7 Department of Urology, West German Cancer Center, University of Duisburg-Essen, University Hospital Essen, Hufelandstr. 55, 45147 Essen, Germany; 8 Szilak Laboratories Bioinformatics and Molecule-Design Ltd., Gem utca 14, H-6723 Szeged, Hungary; laszlo.szilak@gmail.com

**Keywords:** syndecan-1, liver cancer, epithelium, differentiation, shedding, heparan sulfate, Ets-1, AP-1, MMP-7

## Abstract

Syndecan-1 is a transmembrane heparan sulfate proteoglycan which is indispensable in the structural and functional integrity of epithelia. Normal hepatocytes display strong cell surface expression of syndecan-1; however, upon malignant transformation, they may lose it from their cell surfaces. In this study, we demonstrate that re-expression of full-length or ectodomain-deleted syndecan-1 in hepatocellular carcinoma cells downregulates phosphorylation of ERK1/2 and p38, with the truncated form exerting an even stronger effect than the full-length protein. Furthermore, overexpression of syndecan-1 in hepatoma cells is associated with a shift of heparan sulfate structure toward a highly sulfated type specific for normal liver. As a result, cell proliferation and proteolytic shedding of syndecan-1 from the cell surface are restrained, which facilitates redifferentiation of hepatoma cells to a more hepatocyte-like phenotype. Our results highlight the importance of syndecan-1 in the formation and maintenance of differentiated epithelial characteristics in hepatocytes partly via the HGF/ERK/Ets-1 signal transduction pathway. Downregulation of Ets-1 expression alone, however, was not sufficient to replicate the phenotype of syndecan-1 overexpressing cells, indicating the need for additional molecular mechanisms. Accordingly, a reporter gene assay revealed the inhibition of Ets-1 as well as AP-1 transcription factor-induced promoter activation, presumably an effect of the heparan sulfate switch.

## 1. Introduction

Liver cancer is the sixth most commonly diagnosed cancer (782,000 cases/year) and the second most common cause of cancer death (745,000 deaths/year) worldwide [1]. This excessively high mortality to incidence ratio reflects the poor prognosis of liver cancer and underlines the importance of a better understanding of its pathogenesis and biological behavior. Hepatocellular carcinoma (HCC) is a malignant tumor derived from hepatocytes and the most common (75%) histological type of primary liver cancer. Etiological factors include chronic hepatitis B or/and hepatitis C infection, dietary ingestion of aflatoxins, chronic alcohol abuse with subsequent chronic liver disease and cirrhosis, obesity, and diabetes [2] (pp. 205-216). Various signal transduction pathways including Wnt, p53, Rb, Ras, MAPK, JAK/STAT, EGFR/TGF-β, and the heat shock response are commonly altered in liver cancer [3,4].

Syndecan-1 (sdc-1, *SDC1*) is the prototype member of the syndecan family of membrane-bound proteoglycans, featuring heparan sulfate (HS) and chondroitin sulfate chains on its core protein [5,6]. It is predominantly expressed on the basolateral surface of epithelial cells [7]. Normal hepatocytes also express syndecan-1 on their basolateral surface facing the space of Disse [7,8,9,10]. In HCC, the normal sinusoidal and lateral localization pattern of syndecan-1 changes to a more uniform, honeycomb-like membrane distribution, with occasional cytoplasmic and nuclear positivity [9,10,11,12]. Syndecan-1 levels are found upregulated in HCCs that develop in a cirrhotic liver [10]. As a contrast, cell surface syndecan-1 expression is markedly reduced in HCCs with no underlying cirrhosis, especially in those with poor differentiation and high metastatic potential [9,10,13,14].

The intact ectodomains of all mammalian syndecans are constitutively released from cell surfaces by enzymatic cleavage [15,16]. Therefore, shed syndecan-1 is detectable in vitro in conditioned cell culture media (CCM) or in vivo in various body fluids, where its levels often correlate with disease stage. In fact, elevated amounts of shed syndecan-1 can be detected in the sera of patients with HCC as compared with healthy controls, and serum levels of shed syndecan-1 showed a positive correlation with the Barcelona Clinic Liver Cancer staging system, less favorable overall survival, and a greater risk of tumor recurrence and death [17,18]. Despite the abundance of relevant histological data, very little is known about the actual role of syndecan-1 in liver cancer. In the present study, we aimed to address the importance of syndecan-1 in hepatocellular tumor cells, with particular attention to its individual core protein domains.

To date, the exact fate of the post-shedding syndecan-1 remnant is elusive [19,20,21,22,23,24]. Therefore, in addition to full-length syndecan-1, in our model, we include a truncated form of syndecan-1 that contains the intracellular and transmembrane domains, as well as four extracellular amino acids (DRKE motif) but lacks the rest of the extracellular domain. This truncated construct is intended to mimic the potential action of the remnant portion of syndecan-1 after shedding. Because the versatile function of syndecan-1 largely depends on the structure of its heparan sulfate chains, we also attempted to clarify changes in the amount and structure of HS chains upon syndecan-1 transfection.

## 2. Materials and Methods

### 2.1. Cell Lines and Culture Conditions

HepG2 (ATCC HB-8065) [25] and Hep3B (ATCC HB-8064) human hepatocellular carcinoma [26] cell lines were obtained from the American Type Culture Collection (ATCC, Manassas, VA, USA) and maintained in Dulbecco’s Modified Eagle Medium supplemented with 10% fetal bovine serum 1% L-glutamine, and 100 U/mL penicillin-streptomycin. Cell culture consumables were purchased from Sigma-Aldrich Co. (St. Louis, MO, USA) and SARSTEDT AG&Co (Nümbrecht, Germany).

### 2.2. Plasmid Constructs

#### 2.2.1. Mammalian Expression Vectors

Full-length human syndecan-1 cDNA and a deletion mutant, lacking nucleotides coding for amino acids 23–247, i.e., the extracellular domain except the signal peptide and the juxtamembrane DRKE motif, were cloned into mammalian expression vectors pEGFP-N1 and pEGFP-C3 (CLONTECHLaboratories, Inc./BD Biosciences, Palo Alto, CA, USA) and designated “full-length” and “truncated” syndecan-1, respectively (Szilák Laboratories Bioinformatics and Molecule-Design Ltd., Szeged, Hungary), as previously described [20,22]. The host vector, named as “empty vector” (pEGFP-N1) was used as a negative control.

#### 2.2.2. Reporter Vectors

A high affinity consensus Ets-1 response element (ACCGGAAGT)_5_ was designed based on previous reports [27,28,29] and inserted in the promoterless firefly luciferase reporter vector pGL4.16[*luc2CP*/Hygro] (Promega Corporation, Madison, WI, USA). The PathDetect pAP-1-Luc *cis*-reporting plasmid (Agilent Technologies, Santa Clara, CA, USA) contained a firefly luciferase gene under the control of direct repeats of TPA-responsive elements. The pGL4.74[*hRluc*/TK] vector (Promega Corporation) encoding a *Renilla* luciferase gene was used as a control for transfection efficiency.

#### 2.2.3. RNA Interference (RNAi) Expression Vectors

Targeted silencing of Ets-1 expression was achieved using the Block-iT Pol II miR RNAi Expression Vector Kit (Thermo Fisher Scientific, Waltham, MA, USA). Briefly, oligos for microRNAs starting at codons 362 and 641 were designed *in silico* with the Block-iT RNAi Designer software (Thermo Fisher Scientific) (Table S1). Specificity was confirmed by a BLAST search of human RefSeq RNA database. Ligation to pcDNA6.2-GW/EmGFP-miR vector and transformation of One Shot TOP10 chemically competent *Escherichia coli* cells (Thermo Fisher Scientific) were carried out according to the manufacturer’s protocol. Transformants were selected using Blasticidin at 40 μg/mL (Thermo Fisher Scientific). The system produces Emerald green fluorescent protein (EmGFP)-microRNA (miRNA) chimeras selectively targeting *ETS1* transcripts.

#### 2.2.4. Validation of Constructs

All plasmid constructs (empty vector, full-length sdc-1, truncated sdc-1, pEts-1-Luc, pAP-1-Luc, *miR-lacZ, miR-362, miR-641, miR-362+641*) were sequence verified by the Sanger method using the BigDye Terminator v3.1 Cycle Sequencing Kit and ABI PRISM 310 Genetic Analyzer (Thermo Fisher Scientific).

### 2.3. Transfection

HepG2 cells were transfected using Effectene (Qiagen GmbH, Hilden, Germany) nonliposomal lipid reagent as follows. Cells were seeded in 9.5 cm^2^ wells at a density of 3 × 10^5^ cells/well, 24 h prior to transfection. For each well, the transfection mixture contained 0.25 μg plasmid DNA, 100 μL EC buffer, 3.2 μL Enhancer solution, 10 μL Effectene reagent, and 0.6 mL serum-free medium. Hep3B cells were transfected using the Neon (Thermo Fisher Scientific) capillary-type electroporation system. In each well, 5 × 10^5^ cells were mixed with 3 μg plasmid DNA in a 100 μL tip and the following settings were configured: input voltage 1300 V, pulse width 40 ms, and pulse number 1. Using either method, the average transfection efficiencies and viabilities were >70% and >90%, respectively, monitored 24 h post-transfection with confocal laser microscope (Bio-Rad MRC 1024; Bio-Rad Laboratories Inc., Hercules, CA, USA) by the enhanced green fluorescent protein (EGFP) expression and with a microplate reader (VarioskanFlash v4.00.53 using SkanIt Software 2.4.5 RE for Varioskan Flash; Thermo Fisher Scientific) by luciferase activity. Stable transfectants were established by sustained antibiotic selection using the effective concentration of 650 μg/mL G418 (Roche Applied Science, Penzberg, Germany) for HepG2 and 500 μg/mL for Hep3B cells. Stably transfected cultures were enriched for higher expressors using FACSAria High Speed Cell Sorter (Becton-Dickinson, San Jose, CA, USA) and Navios Flow Cytometer (Beckman Coulter, Brea, CA, USA) and Kaluza v1.2 software (Beckman Coulter).

Stably transfected hepatoma cells were examined for any morphological changes. Live cells were observed by phase contrast microscopy (Olympus CK2 inverted phase microscope with an Olympus DP50 CCD camera, Olympus Corporation, Tokyo, Japan); fixed and hematoxylin-eosin (H&E) stained cells were photographed using light microscopy (Olympus BX50 microscope, Olympus Corporation).

### 2.4. Real-Time Quantitative Reverse Transcription PCR (qRT-PCR)

Total RNA was isolated using the Qiagen RNeasy Mini Kit (Qiagen GmbH), according to the instruction of the manufacturer. RNA concentrations were determined with a Nanodrop ND-100 device (Thermo Fisher Scientific). cDNAs were synthesized as follows: 1 μg total RNA, 1 μL 200 ng/μL random primer, and 1 μL 10 mM dNTP mix were incubated in 12 μL final volume, at 65 °C, for 5 min. Subsequently, 4 μL 5x first strand buffer, 2 μL 0.1 M DTT, 1 μL RNase OUT, and 200 U MMLV RT (Thermo Fisher Scientific) were added and the mixture was incubated at 25 °C, for 10 min, then at 37 °C for 50 min. The reaction was inactivated by 5 min incubation at 70 °C.

*SDC1, ETS1,* and *MMP7* mRNAs were detected by real-time PCR amplification on a LightCycler 480 System (Roche Applied Science) using the following program: 95 °C for 10 min, then, 10 touchdown cycles of 95 °C for 30 s, 60 °C with 0.4 °C decrement/cycle for 30 s, 72 °C for 30 s, followed by 40 cycles of amplification at 95 °C for 30 s, 56 °C for 30 s, and 72 °C for 30 s. The reaction mixture included AmpliTaq Gold 360 Master Mix (Thermo Fisher Scientific), ResoLight Dye (Roche Applied Science), specific primers at 200 nM final concentration, and 2 μL cDNA in 10 µL final volume. The examined mRNA expressions were normalized to those of reference genes GAPDH and 18S rRNA (4326317E and 4319413E, Thermo Fisher Scientific). Primer sequences are included in Table S1.

### 2.5. Immunofluorescence

Cells were seeded on glass coverslips and fixed with either 4% *w/v* paraformaldehyde for 10 min or ice-cold methanol for 10 min. Fixed cells were further permeabilized with 0.1% *v/v* Triton X-100 for 10 min when needed. Nonspecific binding was blocked with 5% *w/v* bovine serum albumin (BSA, Merck KGaA, Darmstadt, Germany) and 5% *v/v* nonimmune serum from the host species of secondary antibody. Primary antibodies were applied overnight in 1% *w/v* BSA at 4 °C (Table S2). After washing in PBS, coverslips were incubated with fluorescent-labeled secondary antibodies in 1% *w/v* BSA for 1 h at room temperature (Table S2). Cells were washed and mounted in Vectashield fluorescence mounting medium containing DAPI (Vector Laboratories Inc., Burlingame, CA, USA) or propidium iodide (Merck KGaA). Immunofluorescence images were either captured on a Nikon Eclipse E600 microscope (Nikon Corporation, Tokyo, Japan) connected to the Lucia Cytogenetics v1.5.6 software (Laboratory Imaging, Prague, Czech Republic), or with a Bio-Rad MRC 1024 (Bio-Rad Laboratories Inc.) confocal laser microscope.

### 2.6. Enzyme-Linked Immunosorbent Assay (ELISA)

Cell-bound and shed forms of syndecan-1 were quantified using the CD138 ELISA Kit (Diaclone Research, Besancon, France) according to the instructions of the manufacturer. Conditioned, serum-free media were concentrated using Centricon centrifugal filter devices with 10 kDa nominal molecular weight limit filters (Merck Millipore, Burlington, MA, USA). Cells were lysed in a buffer containing 20 mM Tris-HCl pH 8.0, 150 mM NaCl, 2 mM EDTA, 0.5% *v/v* Triton X-100, and protease inhibitor cocktail (Sigma-Aldrich). The protein content of the samples was determined by Coomassie reagent (Bio-Rad Laboratories Inc.) and 300 μg total protein from each sample was applied to the ELISA plate. Optical densities were read at 450 nm in a LabSystems Multiskan MS microplate reader (Thermo Fisher Scientific).

### 2.7. Western and Dot Blotting

Cells at 80% confluency were harvested in lysis buffer containing 20 mM Tris-HCl (pH 8.0), 150 mM NaCl, 2 mM EDTA, 0.5% *v/v* Triton X-100, protease inhibitor cocktail, 5 mM NaF, 2 mM NaVO_3_ (all Sigma-Aldrich) and centrifuged at 13,000 rpm for 10 min at 4 °C. Supernatants were diluted in Laemmli buffer, boiled, and aliquots containing 25 μg total protein were subjected to sodium dodecyl sulfate polyacrylamide (SDS-PAA) gel electrophoresis. Then, samples were blotted to Immobilon-P PVDF membranes (Merck Millipore) overnight at 4 °C, and blocked with 5% *w/v* BSA, for 1 h, at room temperature. All hardware and reagents for Western blotting were purchased from Bio-Rad Laboratories Inc. For dot blotting, parallel with the control samples, cells were exposed to phorbol ester (phorbol 12-myristate 13-acetate, PMA) at 0.5 μM for 15 min. Two hundred μL CCM per sample was blotted onto the membranes using a Minifold Vacuum Filtration system SRC-96 (Schleicher & Schuell GmbH of Whatman Plc., Dassel, Germany), then subjected to immunoassays. Primary antibodies (Table S2) were applied in 1% *w/v* BSA overnight at 4 °C. Membranes were washed in Tris-buffered saline containing 0.1% *v/v* Tween-20 (TBST), and horseradish peroxidase (HRP)-conjugated secondary antibodies (Table S2) were applied for 1 h at room temperature. After washing in TBST, immunoreactions were visualized by enhanced chemiluminescence using SuperSignal West Pico Chemiluminescent Substrate (Pierce/Thermo Fisher Scientific). Images were obtained using Kodak Image Station 4000MM (Eastman Kodak Company, Rochester, NY, USA) and densitometric analysis was performed using Kodak Molecular Imaging Software 4.0.3. (Eastman Kodak Company) or ImageJ software (Bethesda, MD, USA). For Western and dot blots, chemiluminescent intensities were normalized to Ponceau S staining.

### 2.8. Phospho-Receptor Tyrosine Kinase (pRTK) Array

The phosphorylation status of 42 different RTKs was screened using the Human Phospho-Receptor Tyrosine Kinase Array Kit (R&D Systems, Minneapolis, MN, USA) according to the manufacturer’s protocol (Figure S1). Briefly, confluent monolayers of stable Hep3B transfectants were lysed in lysis buffer containing 1% *v/v* Igepal CA-630, 20 mM Tris-HCl (pH 8.0), 137 mM NaCl, 10% *v/v* glycerol, 2 mM EDTA, 1 mM NaVO_3_, 10 μg/mL aprotinin, 10 μg/mL leupeptin, and 10 μg/mL pepstatin. Lysates were centrifuged at 13,000 rpm for 10 min at 4 °C, and supernatants containing 500 μg total protein were transferred to nitrocellulose membranes containing capture and control antibodies. Following incubation with HRP-conjugated detection antibodies, signals were visualized by ECL, and detected and quantified by Kodak Image Station 4000MM and Kodak Molecular Imaging Software 4.0.3.

### 2.9. Zymography

Twenty μL aliquots of CCM containing 15 μg total protein from each HepG2/Hep3B transfectant were subjected to gelatinase zymogram analysis. Briefly, samples were run on 10% SDS-PAA gels containing 300 μg/mL gelatin. Normal human serum served as a control. Gels were washed in 2.5% Triton X-100 for 30 min and incubated overnight in a solution containing 50 mM Tris (pH 7.5) and 10 mM CaCl_2_ at 37 °C. Then, gels were fixed in 30% methanol/10% acetic acid solution for 30 min and proteinase activities were visualized by Coomassie Blue staining (Bio-Rad Laboratories Inc.). Gelatinase activity appeared as transparent bands against the blue background. Images were taken on a Kodak Image Station 4000MM (Figure S2).

### 2.10. Cell Proliferation Assay

Cell proliferation rates were measured using the Sulforhodamine B (SRB, Sigma-Aldrich) colorimetric assay [30]. In brief, cells were counted using a hemocytometer and Trypan Blue dye exclusion and seeded in 96-well plates at a density of 5 × 10^3^ cells/well in 100 μL culture medium. Each transfectant, at each time point, was represented in at least 8 parallel wells. Every 24 h, cells were fixed with 10% trichloroacetic acid for 1 h, at 4 °C. After an extensive wash with tap water, plates were allowed to dry, and were stained with 0.4% SRB in 1% acetic acid for 30 min. Following extensive wash with 1% acetic acid, plates were dried, and the dye was dissolved in 10 mM Tris-HCl. Color intensity was measured at 570 nm absorbance using a LabSystems Multiskan MS microplate reader. Doubling times were calculated from the log-phase of growth curves.

### 2.11. Statistical Analysis

Data were analyzed using Microsoft Excel v.2016 (Microsoft Corp., Redmond, WA, USA) and GraphPad Prism 7 (GraphPad Software, La Jolla, CA, USA). Data from syndecan-1 transfectants were normalized to those containing the empty vector, miR transfectants were normalized to those expressing *miR-lacZ*. For ELISA, qRT-PCR, luciferase reporter assay, pRTK array, Western and dot blot, the data from empty vector vs. sdc-1 transfected forms and from *miR-lacZ* vs. *miR-362*, *miR-641*, and *miR-362+641* transfected forms were analyzed by unpaired Student’s *t*-test. For dot blot, data from full-length vs. truncated sdc-1 transfectants were analyzed by unpaired Student’s *t*-test. Statistical significance was considered at *p* < 0.05.

## 3. Results

### 3.1. Expression of Wild-Type and Truncated Syndecan-1 in Hepatoma Cells

**Figure 1:**
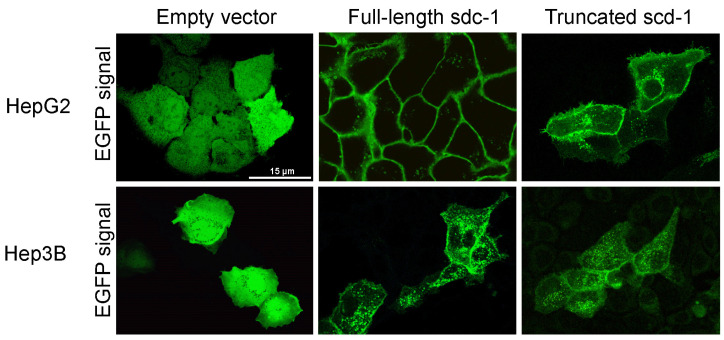
Immunofluorescence images of HepG2 and Hep3B cells after transfection with empty enhanced green fluorescent protein (EGFP) vector, as well as full-length and truncated syndecan-1 EGFP constructs. A diffuse green signal was seen after transfection with the empty vector. Upon transfection with the full-length syndecan-1 construct, the fluorescent signal localized to the cell membrane. Although truncated syndecan-1 partly localized to the cytoplasm, the signal was enriched at the plasma membrane, indicating that truncated syndecan-1, lacking the extracellular domain, was sorted successfully. Representative images are at 1000× magnification (scale bar 15 μm).

Full-length and truncated syndecan-1 were transfected into HepG2 and Hep3B cells. Stable transfectants were established and subcellular localization of the fusion protein products was observed by fluorescent microscopy (Figure 1). Full-length syndecan-1 localized primarily to the plasma membrane and to a lesser extent to cytoplasmic vesicular structures. Truncated syndecan-1 showed a weaker fluorescent signal on the cell surface and was more abundant in the endosomal compartment. Notably, both full-length and truncated syndecan-1 constructs preferentially localized to the plasma membrane at areas of cell-cell contact. Diffuse green fluorescence was detected in the empty vector transfected cells.

### 3.2. Overexpression of Full-Length or Truncated Syndecan-1 Induces a Hepatocyte-Like Cell Morphology

**Figure 2:**
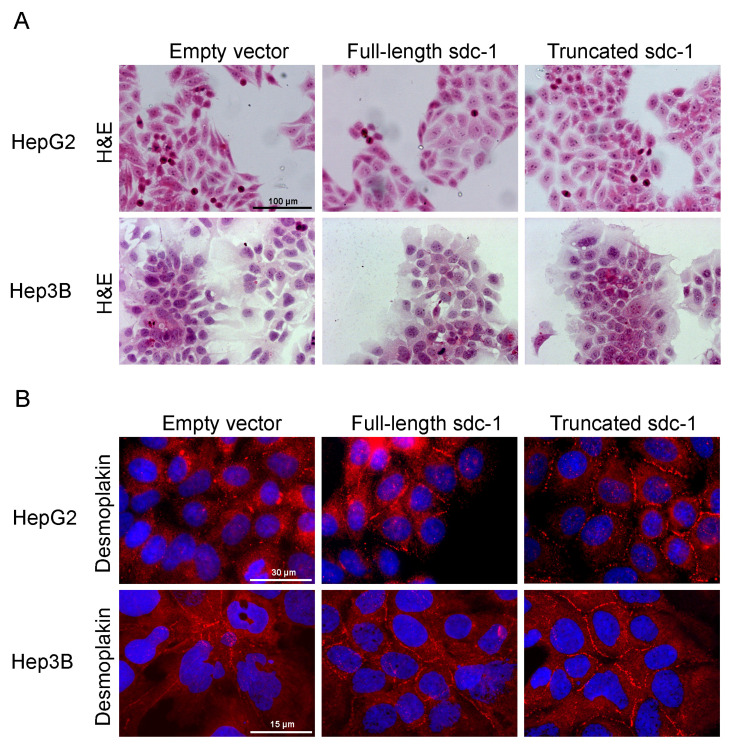

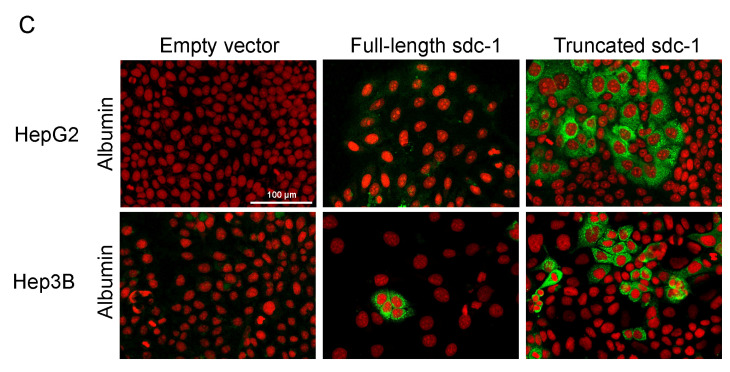
Hepatocyte-like differentiation of HepG2 and Hep3B cells upon overexpression of full-length or truncated syndecan-1. (**A**) H&E stained empty vector transfected control cells were oval or spindle shape with prominent nuclei and high nucleus-to-cytoplasm ratio, and featured frequent cell divisions. In the syndecan-1-transfected cell lines, the numbers of poorly differentiated and dividing cells decreased, and groups of cells with a more hepatocyte-like morphology appeared. Cells showing signs of differentiation were characterized by a lower nucleus-to-cytoplasm ratio; (**B**) As a sign of cell differentiation, desmoplakin immunocytochemistry showed relocalization of the protein to the cell surface in syndecan-1-transfected cells. In the native hepatoma cell lines, desmoplakin mainly localized to the cytoplasm. Red, desmoplakin and blue, nuclei; (**C**) Numerous albumin-positive cells were detected among the truncated syndecan-1 transfectants. Green, albumin and red, nuclei. Representative images are at 1000× magnification (scale bar 15 μm), 600× magnification (scale bar 30 μm), and 200× magnification (scale bar 100 μm).

HepG2 and Hep3B cells were selected as transfection targets; as HepG2 expressed, whereas Hep3B contained hardly any endogenous syndecan-1, thus, we presumed that they would respond differently to syndecan-1 transfection. The morphology of the transfected cell lines was examined by H&E staining. Remarkably, overexpression of full-length or truncated syndecan-1 resulted in morphological signs of hepatocyte-like differentiation. Typical features of hepatomas such as heterogeneous cell size, irregular nuclear staining, elongated cell shape, and the presence of dividing cells, were partially replaced by a more homogenous, isodiametric morphology. A cobblestone pattern was more prominent in HepG2 transfectants, while the disappearance of the fusiform component from cultures was characteristic of Hep3B transfectants. In low-confluence cultures, syndecan-1 overexpressing cells spread better and had a decreased nuclear-to-cytoplasmic ratio. In near-confluent and confluent cultures, however, syndecan-1 overexpressing cells tended to form denser islets. The number of dividing cells also decreased markedly in the areas displaying differentiation (Figure 2A). The above changes in cell morphology were more prominent in hepatoma cells overexpressing the truncated form of syndecan-1.

Cells were immunostained for desmoplakin, an obligate component of functional desmosomes. As opposed to cells transfected with the empty vector where desmoplakin was dispersed in the cytoplasm, syndecan-1 transfected cells recruited desmoplakin to areas of cell-cell contact serving as a marker of cell differentiation (Figure 2B). Importantly, albumin expression was reactivated in truncated syndecan-1 transfectants, as observed by fluorescent immunocytochemistry (Figure 2C). Changes in cell morphology, as described above, appeared more markedly in hepatoma cells overexpressing the truncated form of syndecan-1.

### 3.3. Syndecan-1 Status of the Transfectant Cells

**Figure 3:**
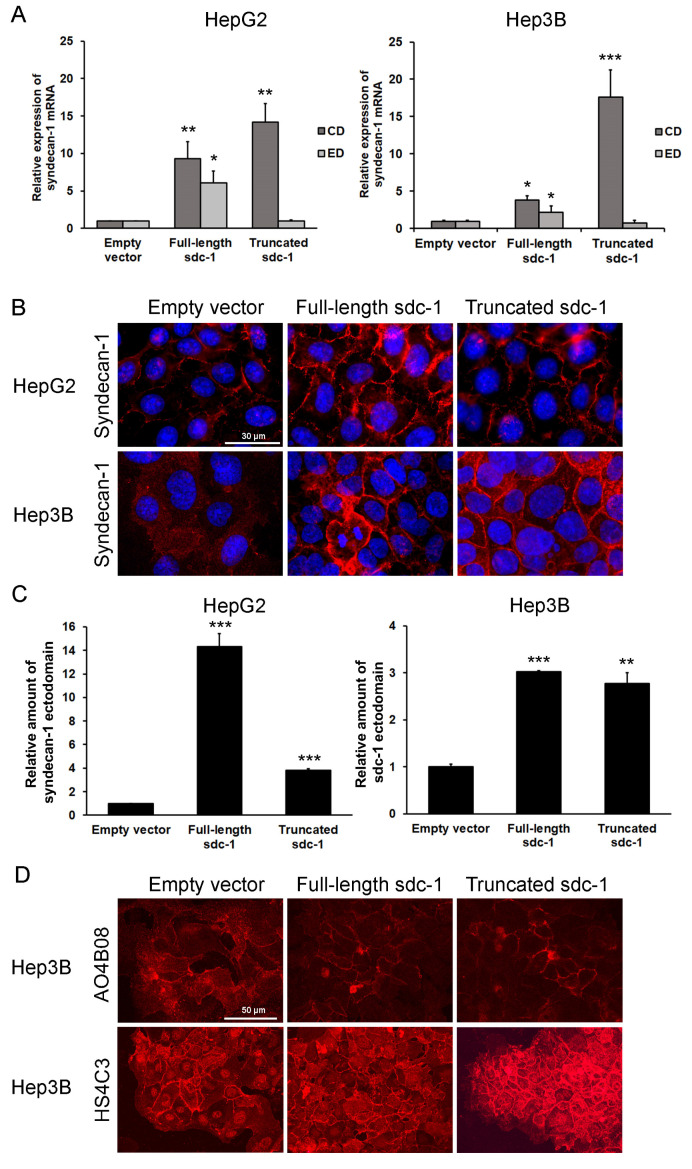
Syndecan-1 mRNA and protein levels in the empty vector and syndecan-1 transfected HepG2 and Hep3B cell lines. (**A**) Transcripts coding for the extracellular domain (ED) and the cytoplasmic domain (CD) of syndecan-1 were elevated in the full-length transfectants, while the expression of the CD only was increased in the truncated transfectants, whereas that of the ectodomain-coding transcript remained unchanged; (**B**) High amounts of full-length (ectodomain-containing) syndecan-1 protein were detected on the surface of both full-length and truncated syndecan-1 transfectants by immunofluorescence using an ectodomain-specific antibody; (**C**) ELISA confirmed elevation of full-length syndecan-1 in both transfectants; (**D**) A comparison with the empty vector-transfected cells, shows that the abundance of AO4B08-reactive (internal 2-*O*-sulfated/6-*O*-sulfated) HS epitopes are decreased, whereas the amount of HS4C3-reactive (highly sulfated, 3-*O*-sulfated) epitopes are increased in both Hep3B cell lines containing syndecan-1 constructs. Data points represent the mean ± standard deviation (SD), n = 3; * *p* < 0.05; ** *p* < 0.01; *** *p* < 0.001 versus the empty vector control. Representative images are at 600× magnification (scale bar 30 μm) and 400× magnification (scale bar 50 μm).

To elucidate the mechanism by which the overexpression of full-length or truncated syndecan-1 resulted in the phenotypical changes observed, we next evaluated whether and how syndecan-1 turnover was affected in our cells. As expected, mRNA coding for the cytoplasmic domain of syndecan-1 was elevated in both full-length and truncated syndecan-1 transfected cells, whereas mRNA coding for the syndecan-1 ectodomain was elevated only in the full-length transfected cells (Figure 3A). Surprisingly, however, syndecan-1 ectodomain protein levels were increased in both transfectants, as shown by immunofluorescence and ELISA using a syndecan-1 ectodomain-specific antibody (Figure 3B,C). To elucidate the mechanism regarding why endogenous syndecan-1 was expressed in truncated syndecan-1 expressing cells, further experiments were carried out.

### 3.4. Shift of Heparan Sulfate Epitope Abundance in Syndecan-1-Transfected Hep3B Cells

Heparan sulfate structure was probed using two structure-specific heparan sulfate antibodies, HS4C3 and AO4B08. HS4C3 recognizes highly sulfated and 3-*O*-sulfated motifs, whereas AO4B08 reacts with epitopes possessing an internal 2-*O*-sulfated iduronic acid residue and more than one 6-*O*-sulfate groups [10,31,32,33]. The intensity of AO4B08 immunoreaction was reduced upon transfection with either full-length or truncated syndecan-1 as compared with the empty vector control; in contrast, HS4C3-reactive epitopes were markedly upregulated in both transfectants, especially in those expressing the truncated variant. In addition to the cell surface, HS4C3 was also clearly detected in cell nuclei (Figure 3D). Taken together, heparan sulfate modifications were shifted in syndecan-1 transfected cell lines from AO4B08-reactive towards HS4C3-reactive epitopes. Of note, the empty vector-transfected cells also contained low amounts of HS4C3-reactive heparan sulfate which, however, was probably linked to another proteoglycan core, as syndecan-1 was hardly expressed in these cells.

### 3.5. Syndecan-1 Shedding is Inhibited by Truncated Syndecan-1 in Hepatoma Cells

**Figure 4:**
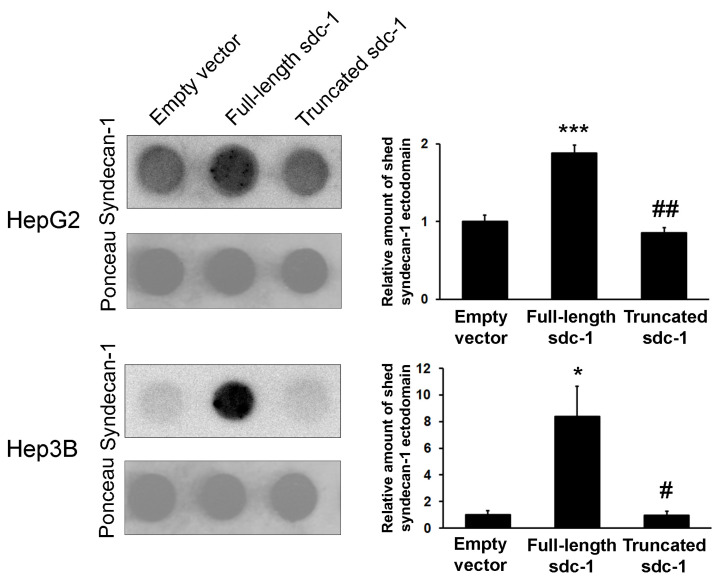
Shedding of syndecan-1 ectodomain. The amount of shed (soluble) syndecan-1 ectodomain was measured from cell culture media (CCM) by ectodomain-specific antibody. Shedding of the ectodomain was increased in full-length transfectants, whereas it remained unchanged in truncated transfectants in spite of the overall increased abundance of endogenous syndecan-1 (see Figure 3B). Data points represent the mean ± SD, n = 2; * *p* < 0.05; *** *p* < 0.001 versus the empty vector transfectant; ^#^ *p* < 0.05; ^##^ *p* < 0.01 versus the full-length sdc-1 transfectant.

The elevated syndecan-1 ectodomains on the surface of the truncated syndecan-1 expressing cells could indicate an enhanced translation or extended half-life of endogenous syndecan-1 molecules, the latter being a result of decreased proteolytic shedding of endogenous syndecan-1 from the cell surface. To test this hypothesis, we performed a dot blot assay to quantify the presence of shed syndecan-1 protein ectodomains in the CCM of our cell lines (Figure 4). A comparison with the empty vector-transfected cells showed that the amount of shed syndecan-1 ectodomain was increased in the CCM of full-length syndecan-1 transfectants; however, despite an increased abundance of the native protein on the cell surface as demonstrated by immunofluorescence, ELISA and syndecan-1 ectodomain shedding remained as low as the control, in the CCM of truncated syndecan-1 overexpressing cells. To further demonstrate that endogenous syndecan-1 was enhanced because of its suppressed shedding in truncated transfectants, cells were treated with PMA that induced shedding (Figure S3). Indeed, the amount of shed syndecan-1 ectodomain in the CCM of truncated transfectants was greatly increased by PMA.

### 3.6. Truncated Syndecan-1 Suppresses Proliferation of Hepatoma Cells

**Figure 5:**
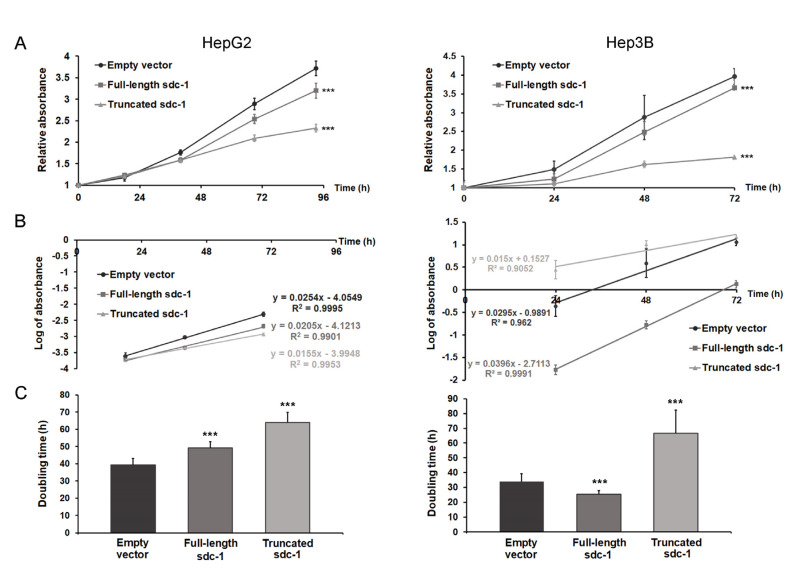
The effect of syndecan-1 overexpression on the proliferation of HepG2 and Hep3B cell lines. (**A**) Transfection with the truncated syndecan-1 construct significantly slowed down the proliferation of both HepG2 and Hep3B cell lines. A moderate decrease in the proliferation of HepG2 full-length transfectant was also observed. Notably, overexpression of full-length syndecan-1 in Hep3B cells led to a decrease in double time from 33.89 to 25.27 h (by 25.4%); (**B**) The log phase of growth curves; (**C**) Doubling time calculated from the log phase of growth curves. Data points represent the mean ± SD, n = 8; *** *p* < 0.001 versus the empty vector transfectant.

The proliferation rate of truncated syndecan-1 transfectants decreased significantly in both hepatoma cell lines (Student’ *t*-test, HepG2 *p* < 0.001 and Hep3B *p* < 0.001) (Figure 5A,B). In HepG2, the generation time increased from 39.4 to 49.2 h (by 24.9%) and to 64 h (by 62.7%) in full-length and truncated syndecan-1 transfectants, respectively, whereas in Hep3B, it was increased from 34.6 to 52.4 h (by 51.2%) in truncated syndecan-1 transfectants as compared with the empty vector control. For both cell lines, the difference between control and truncated transfectants was significant at *p* < 0.001 (Student’ *t*-test) (Figure 5C). Transfection of Hep3B with full-length syndecan-1 appeared to reduce doubling time to 30.2 h (by 12.9%); whereas this difference was statistically significant, its biological relevance was probably minor (Figure 5C).

### 3.7. Overexpression of Truncated Syndecan-1 Downregulates MMP-7 Expression in HCC Cells

**Figure 6:**
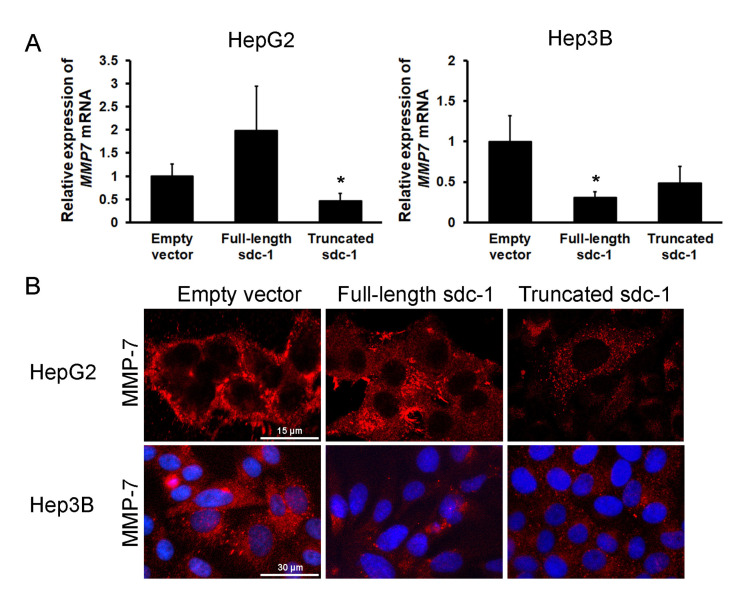
The effect of syndecan-1 constructs on MMP-7 expression. (**A**) Except for HepG2 transfected with full-length syndecan-1, all other syndecan-1 transfectants displayed decreased *MMP7* mRNA expression and (**B**) protein levels as compared with the empty vector transfected cells. Red, MMP-7 and blue, nuclei. Data points represent the mean ± SD, n = 3; * *p* < 0.05 versus the empty vector transfectant. Representative images are at 1000× magnification (scale bar 15 μm) and 600× magnification (scale bar 30 μm).

In vivo, proteolytic shedding of syndecan-1 ectodomains was shown to be mediated by MMP-7 [34,35]; therefore, we evaluated MMP-7 expression in our cells (Figure 6). Except for HepG2 transfected with full-length syndecan-1, decreased expression of MMP-7 was detected in syndecan-1 transfectants by qRT-PCR and immunostaining. Zymography also corroborated the decreased activity of MMP-7 protease in full-length syndecan-1 Hep3B transfectants. The MT-MMP-1 (MMP-14) could have compensated for the decreased amount of MMP-7 (Figure S2).

### 3.8. Signaling Pathways Converging to Ets-1 Are Downregulated in Hepatoma Cells Overexpressing Full-Length or Truncated Syndecan-1

**Figure 7:**
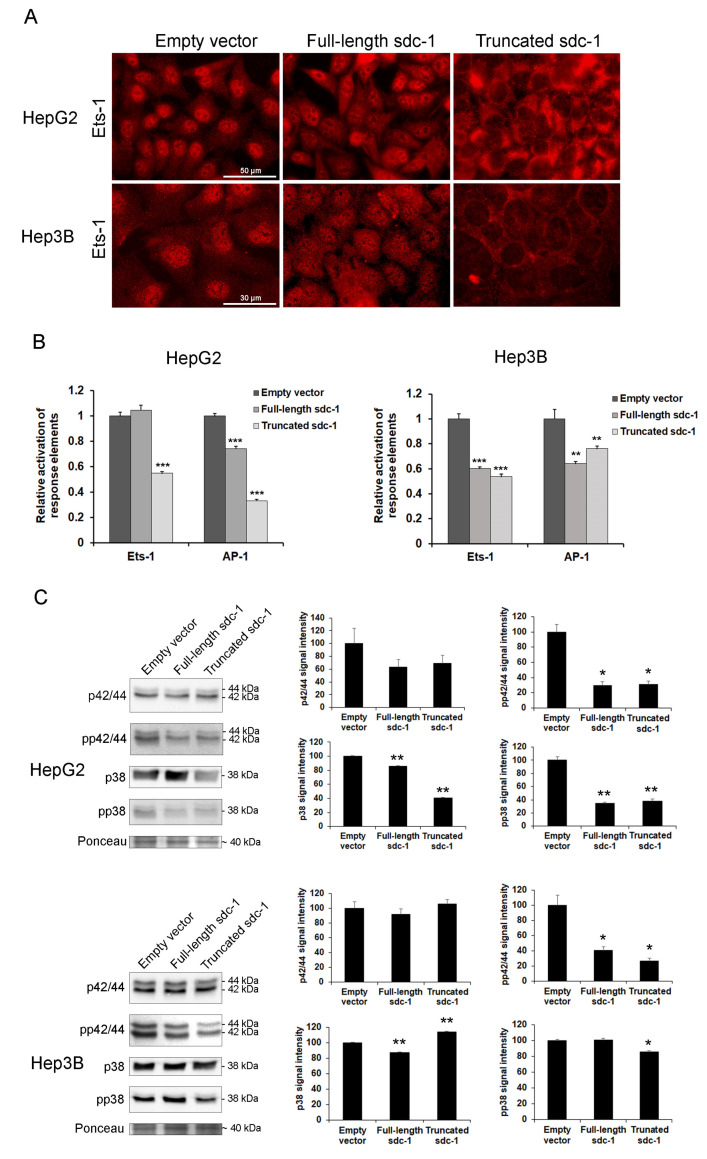
Decreased activity of Ets-1 and AP-1 upon syndecan-1 overexpression. (**A**) Intensive nuclear Ets-1 immunopositivity was present in the empty vector-transfected hepatoma cell lines. A modest decrease of the staining intensity was found in the full-length syndecan-1 transfected cells, whereas nuclei of truncated syndecan-1 transfectants showed hardly any Ets-1 immunoreaction. The protein was partially (full-length) or totally (truncated) sequestered in the cytoplasm; (**B**) In HepG2, decreased Ets-1 response element-driven promoter activity was found in the truncated transfectants, whereas AP-1 response element-driven promoter activity was suppressed in both full-length and truncated syndecan-1 cell lines. In Hep3B, both syndecan-1 constructs interfered with Ets-1 and AP-1 response element-driven promoter activity; (**C**) Transfection with either syndecan-1 construct inhibited the activation of ERK1/2 and p38 MAP kinases. Data points represent the mean ± SD, (B) n = 3, (C) n = 2; * *p* < 0.05; ** *p* < 0.01; *** *p* < 0.001 versus the empty vector transfectant. Representative images are at 600× magnification (scale bar 30 μm) and 400× magnification (scale bar 50 μm).

Expression of various MMPs is typically regulated by the ETS and AP-1 families of transcription factors, therefore, we evaluated their expression at both protein and functional activity levels using immunocytochemistry, and luciferase reporter assays (Figure 7A,B). Although the amount of Ets-1 did not seem to change significantly, its nuclear localization diminished markedly in the truncated syndecan-1 transfectants, although the cytosol was saturated with Ets-1 (Figure 7A). The relative activation of Ets-1 response elements did not decrease in the full-length syndecan-1 transfected HepG2 cells, whereas all the other response elements we studied were downregulated as compared with the empty vector containing control cell lines. This could explain why MMP-7 expression remained invariant in the full-length syndecan-1 transfected HepG2 cell line (Figure 6). In the meantime, in the case of AP-1 response elements, all transfected cell lines showed decreased activity (Figure 7B).

Since the Ets-1 transcription factor is known to be activated by ERK1/2 and p38 kinases, we also measured protein levels and phosphorylation status of these proteins using Western blot. Both the amounts and activating phosphorylations of ERK1/2 and p38 (T202/Y204 and T185/Y187 on ERK1 and ERK2, respectively; T180 and Y182 on p38) tended to diminish upon syndecan-1 overexpression, with some changes being significant (Figure 7C).

### 3.9. Silencing of Ets-1 Alone is Insufficient to Induce Epithelial Differentiation in Hepatoma Cell Lines

**Figure 8:**
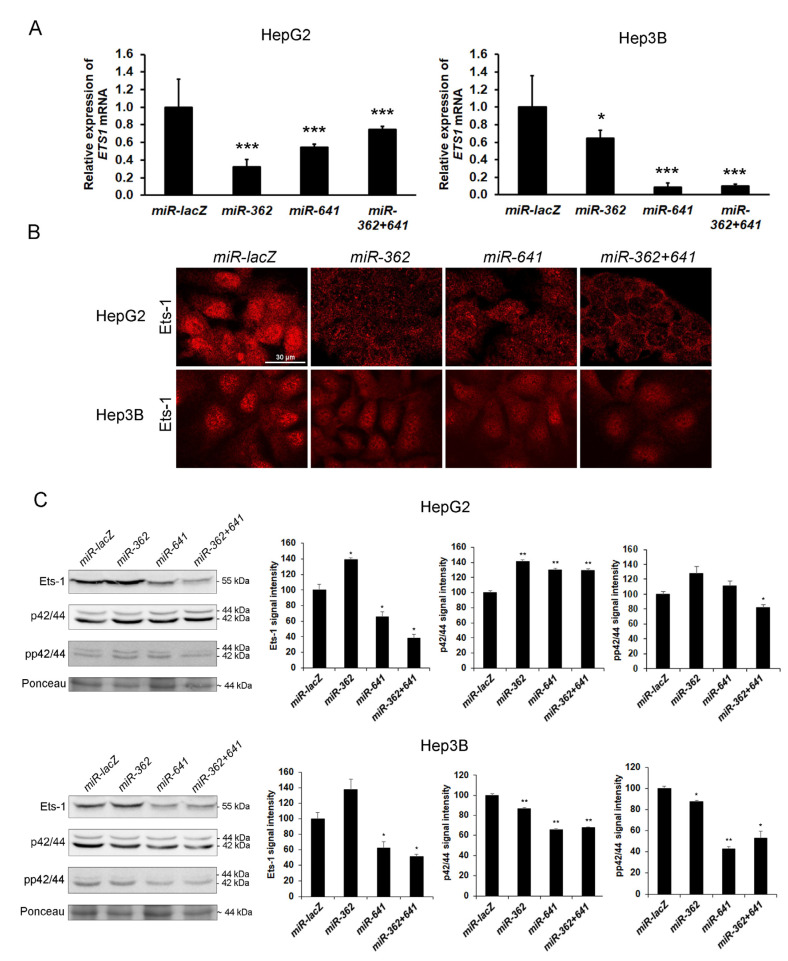
Silencing of Ets-1 by RNA interference in HepG2 and Hep3B cells. (**A**) As indicated by the qRT-PCR results, knockdown of Ets-1 expression was successful in both cell lines; (**B**) The silencing constructs effectively hindered Ets-1 protein expression; (**C**) Except for *miR-362*, Western blots confirmed the downregulation of Ets-1; however, inactivation of ERK1/2 was mostly detected in Hep3B cell line. Data points represent the mean ± SD, (A) n = 3, (C) n = 2; * *p* < 0.05; ** *p* < 0.01; *** *p* < 0.001 versus *miR-lacZ* transfectant. Representative images are at 600× magnification (scale bar 30 μm).

To confirm that syndecan-1 induced downregulation of Ets-1 is in a causal relationship with hepatocyte-like differentiation of the transfected hepatoma cells, wild-type HepG2 and Hep3B cell lines were stably transfected with miRNA expressing plasmids, i.e., designated *miR-362, miR-641* and *miR-362+641*, targeting *ETS1* transcripts. Control cells were transfected with a miRNA targeting *lacZ* transcripts (*miR-lacZ*). Successful knockdown of Ets-1 was confirmed by fluorescent immunocytochemistry, qRT-PCR, and Western blot (Figure 8).

**Figure 9:**
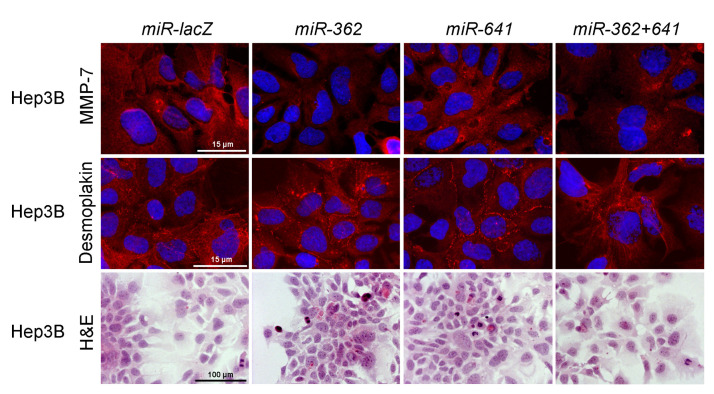
Changes in cell morphology upon Ets-1 silencing. With regard to inhibition of MMP-7 and differentiation, miRNAs targeting Ets-1 failed to fully replicate the changes observed after syndecan-1 transfection. The *miR-641* did not inhibit the expression of MMP-7 and only modest desmoplakin relocalization was detected. H&E staining showed very few differentiated cells throughout the various transfectants. Representative images are at 1000× magnification (scale bar 15 μm) and 200× magnification (scale bar 100 μm).

Unlike the overexpression of syndecan-1 that inhibited *MMP7* in Hep3B cells, downregulation of *ETS1* alone by miRNA did not result in consistent suppression of MMP-7. While some of the effects of syndecan-1 were reproduced, for example, desmoplakin localization was moderately redistributed to areas of cell-cell contacts, as seen in Hep3B cells, and epithelial cell morphology was partially regained, *ETS1* silencing failed to fully replicate the syndecan-1 induced phenotype shift (Figure 9).

## 4. Discussion

On the basis of in vitro observations on mouse mammary tumor cells [36], Jalkanen and colleagues suggested that the loss or suppression of syndecan-1 expression had an important role in driving malignant transformation, as restitution of syndecan-1 expression cells regained epithelial morphology and diminished anchorage-independent malignant growth [37]. The degree of syndecan-1 loss in carcinomas often correlates with histological grade and indicates poor prognosis, as shown in head and neck [38,39,40], hepatocellular [13,14], colorectal [41,42,43,44], cervical [45], pancreatic [46], renal carcinomas [47], and bladder [48] cancers. Intriguingly, strong expression of syndecan-1 can also confer aggressive behavior to some tumor types as reported in dedifferentiated thyroid [49], poorly differentiated prostate [50,51], high grade breast [52,53], high grade ovarian [54,55], and high grade hepatocellular carcinomas [12].

Syndecan-1, as a major proteoglycan constituent of the liver, is extensively implicated in the physiological function of hepatocytes. Liver diseases are often accompanied by quantitative changes of syndecan-1. Liver cirrhosis is characterized by increased amounts of syndecan-1, and shed syndecan-1 has been experimentally shown to protect against fibrotic remodeling [56]. The significance of quantitative changes of syndecan-1 in liver cancer is still not clearly understood [57]. In the present study, we made an effort to investigate the importance of syndecan-1 in HCC, where loss of syndecan-1 was frequently observed and associated with poor prognosis. Therefore, we restored the expression of full-length or ectodomain-deleted syndecan-1 in two HCC cell lines.

In line with previous reports [58], syndecan-1 was expressed moderately in HepG2, and hardly expressed in Hep3B cells. The full-length and truncated syndecan-1 constructs were both successfully expressed and showed enrichment in the plasma membrane, mostly at sites of cell-cell contact. This observation implies that the ectodomain is dispensable for the cell surface localization of truncated syndecan-1. However, it is to be noted that our truncated syndecan-1 construct still contained the transmembrane domain and membrane-proximal four amino acids of the ectodomain that were found important for the dimerization [20,59,60].

Overexpression of syndecan-1, especially of the truncated construct, resulted in a more differentiated and hepatocyte-like phenotype. This change was accompanied by enhanced formation of desmosomes, as indicated by the accumulation of desmoplakin at sites of cell-cell contact. Desmoplakin provides the link to intermediate filaments, and therefore is an obligate component for the stabilization of desmosomes and, consequently, tissue architecture [61]. Accordingly, abnormal localization and loss of desmoplakin were shown to correlate with progression in several types of cancer [62] including HCC [63]. Overexpression of truncated syndecan-1 also triggered albumin production and reduced cell proliferation, both of which were benchmarks of a more differentiated phenotype.

Transfection of B6FS fibrosarcoma and STAV-AB malignant mesothelioma cells with the same two constructs used in this study also resulted in decreased proliferation and a more epithelioid cell morphology [20]. On the contrary, both constructs decreased doubling time in HT-1080 fibrosarcoma cells [22]. A recent study demonstrated the interaction of syndecan-1 with important regulatory proteins inside the nucleus [64].

Unexpectedly, immunostaining revealed an elevated amount of full-length syndecan-1 on the cell surface of cells transfected with truncated syndecan-1. Expression of the truncated form did not enhance the transcription of the endogenous syndecan-1, however, it could have increased its translation or inhibited the shedding of its ectodomain. Overexpression of full-length syndecan-1 increased the amount of shed ectodomains, which aligned with previous findings [65]. Although matrix metalloproteinases with syndecan-1 sheddase activity are primarily produced by stromal cells [34,35,66,67,68,69], MMP-7 and MMP-14 can be expressed by normal, as well as tumorous hepatocytes [56,70]. We found that MMP-7 was suppressed in cells overexpressing the truncated form of syndecan-1. It is plausible that transfection with ectodomain-deleted syndecan-1 mimics the post-shedding membrane stub and exerts feedback inhibition towards MMP-7 production. Multiple mechanisms that could mediate such regulation have been published. Activation of MAPK, EGF, and PKCα can facilitate syndecan-1 shedding. Our experiments revealed that PMA exposure enhanced syndecan-1 shedding, which indicated the potential implication of PKCα [71].

The fate of truncated syndecan-1 requires more investigation. In a process termed regulated intramembrane proteolysis (RIP), the remaining core protein stub could be subjected to intramembrane cleavage, where an intramembrane-cleaving protease would release the cytoplasmic domain without membrane damage, rendering it available for proteasomal degradation or translocation to the nucleus [72,73,74]. RIP presumably occurs only if the remaining ectodomain stub is shorter than 30 amino acid residues [75]. In our truncated syndecan-1 construct, the truncated syndecan-1 core protein was extracellularly tagged with EGFP, therefore, it could not be subject to RIP. It looks plausible that EGFP protects the protein from intramembrane proteolysis, thus, rendering it resistant to RIP.

To elucidate further mechanisms by which truncated syndecan-1 suppressed syndecan-1 shedding, we examined transcriptional regulation of *MMP7* and its connection to the MAPK signaling pathway, actively involved in the control of syndecan-1 shedding [71,76]. The promoter region of *MMP7* contains AP-1 and PEA3 motifs, which serve as binding sites for complexes composed of members of the JUN, FOS, and ETS families of transcription factors [70,77,78]. We confirmed downregulation of the activity of promoters containing Ets-1 or AP-1 binding sites, as well as decreased Ets-1 protein expression, in cells overexpressing truncated syndecan-1. In fact, Ets-1 expression has also been known to be induced by AP-1 and Ets-1 itself [79,80], and thus this loop appeared to be downregulated in our system. Promoter-binding activity of Ets-1 has been shown to be controlled by several molecules including ERK1/2 [81] and p38 [82]. We could indeed confirm decreased amounts of phosphorylated p38 and phosphorylated ERK1/2 in cells transfected with truncated syndecan-1.

We have previously described that HS of liver origin interfered with DNA binding of Ets-1 and AP-1, indicating that it was also capable of competing with the transcription initiated by these transcription factors [83]. Transfection with truncated syndecan-1 and, to a lesser extent, with full-length syndecan-1, brought about a shift in HS epitope abundance from AO4B08-reactive epitopes, previously shown to be upregulated in HCC [10], in favor of liver-specific HS4C3-reactive epitopes. Furthermore, immunoreaction clearly localized this epitope in the nuclei of tumor cells, as well. Thus, we speculated that downregulation of ETS1 and AP1 activity, confirmed by reporter gene assay, could be at least in part, related to the inhibitory action of the HS4C3-reactive HS chains of syndecan-1 [84]. As wild-type Hep3B cells only express trace amounts of syndecan-1, HS detected in these cells could belong to proteoglycans other than syndecan-1. Although more detailed analysis of HS structure was beyond our technical capabilities, we believe, in line with recently published data [85,86], that the versatile function of syndecan-1 highly depends on the structure of its sugar chains, especially its HS epitopes. HS chains engage in selective interactions with growth factors and cytokines, endowing syndecans with a plethora of functions, greatly influenced by the amount and location of sulfation, many of which are yet to be discovered.

Another mechanism to inhibit *ETS1* transcription is related to the downregulation of HGFR (c-Met). It has been published that exposure of hepatoma cell lines to HGF induced transcription of ETS1 and, subsequently, MMPs, whereas silencing of *ETS1* downregulated production of MMPs [87]. Therefore, the reduction in Ets-1 levels observed in our system could ultimately be caused by the downregulation of the HGF/ERK/Ets-1 pathway [76]. This hypothesis is supported by our preliminary results using a phosphorylated receptor tyrosine kinase array that showed a marked reduction in HGFR activation in syndecan-1 transfected Hep3B cells. Interestingly, *MET* itself is transcriptionally upregulated by Ets-1 and, in turn, activation of c-Met induces transcription of *ETS1* showing that Ets-1 can act both upstream and downstream of c-Met (Figure S2) [87,88].

Ets-1 has been shown to be involved in a variety of processes, such as cell differentiation, proliferation, tissue remodeling, angiogenesis, apoptosis, transformation, and metastasis [89]. In a previous study, we found a decrease in the expression of syndecan-1 and opposite trend for Ets-1 throughout colon carcinoma progression [90]. In HCC, increased transcriptional activity of *MMP7* and *ETS1* as compared with adjacent noncancerous liver tissue [78] statistically correlated with tumor progression of HCCs [91].

To confirm the implication of downregulated Ets-1 in the partial differentiation and suppressed proliferation of hepatoma cells overexpressing syndecan-1, we knocked down Ets-1 expression by miRNAs. The effect of miR inhibition was more pronounced in Hep3B cells, where reduced levels of Ets-1 partially replicated the effects of syndecan-1 overexpression (e.g., a trend towards differentiation), indicating the implication of Ets-1 in these events. However, since the effect of *ETS1* silencing was only partial as compared with syndecan-1 overexpression, we speculated that there was a need for other factors, such as AP-1, that could also mediate the effect of overexpressed syndecan-1. AP-1 transcription factors are of particular importance in liver cancer. Conditional inactivation of c-Jun in the liver reduces the proliferative capacity of hepatocytes after partial hepatectomy [92] and interferes with the development of liver tumors [93]. HCCs are also characterized by a significantly higher c-Fos expression as compared with non-tumor tissues [94]. In agreement with literature data, we found decreased AP-1 promoter-binding activity upon overexpression of truncated syndecan-1.

A limitation to our in vitro model is the lack of stromal components, which could significantly influence the behavior of the tumor tissue. Ectopic expression of syndecan-1 in stromal cells has been reported in carcinomas of the cervix [95,96], and stromal syndecan-1 expression was shown to correlate with poor prognosis in some tumor types such as cancers of the stomach [97], pancreas [46], endometrium [98], oral cavity [99], breast [100,101], bladder [48], and ovary [102].

**Figure 10:**
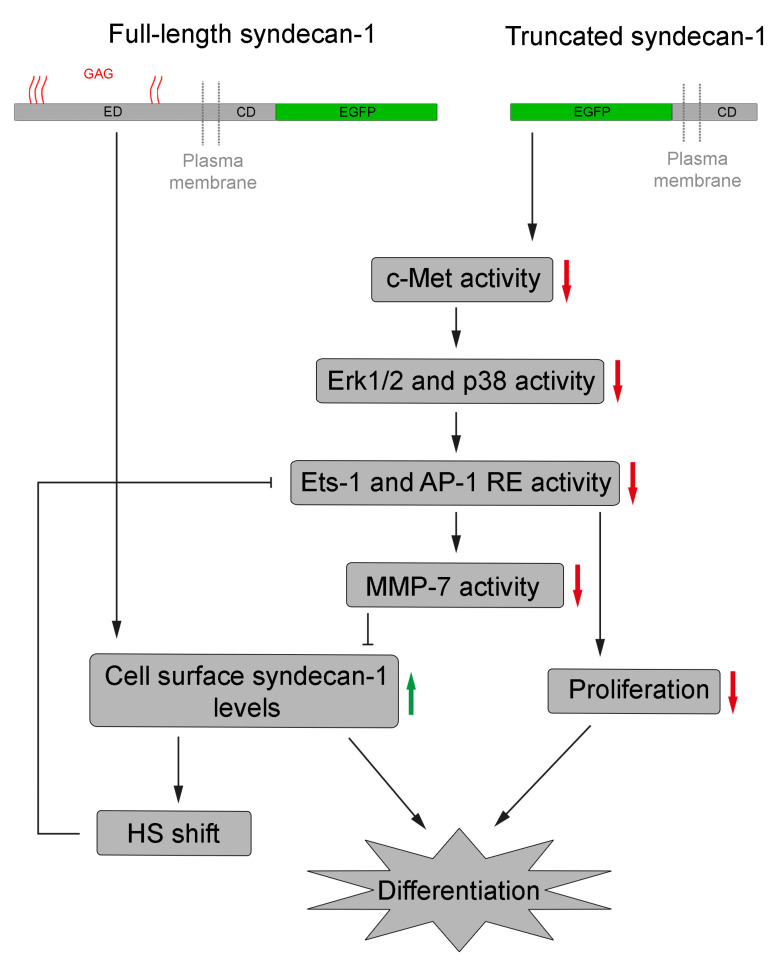
A schematic model to explain the effects of syndecan-1 overexpression. The central event is the accumulation of intact syndecan-1 molecules on the cell surface. This is achieved directly in cells transfected with the full-length construct and indirectly in cells transfected with the truncated syndecan-1 construct. The downstream effect is reduced Ets-1- and AP-1-mediated promoter activation with subsequent inhibition of cell proliferation and differentiation. RE, response element and GAG, glycosaminoglycan.

Future studies are needed to complement our results by determining additional effects of syndecan-1, either full-length or truncated, on the phenotype and behavior of hepatoma cells. Such research is not unprecedented; for instance, the importance of syndecan-1 in hepatoma cell migration and invasion has been demonstrated [58,103], and the role of certain miRNAs in the negative regulation of both syndecan-1 [104] and Ets-1 [105] expression has been described, albeit the root causes of these expressional changes during tumor progression remain to be elucidated in more detail (Figure 10).

## 5. Conclusions

While syndecan-1 is known to play a key role in the normal function of the liver, its participation in the development and progression of HCC is not clarified. To address this question, two HCC cell lines with decreased syndecan-1 expression were transfected with the full-length or truncated forms of syndecan-1. The latter can mimic the situation that occurs after ectodomain shedding. Overexpression of syndecan-1, especially in its truncated form, slowed down the proliferation and induced differentiation of HCC cells. The molecular mechanisms implicated in this effect included decreased MAPK signaling and a shift in the relative abundance of various HS epitopes. Both pathways converged on decreased nuclear availability of the Ets-1 transcription factor which is known to stimulate the transcription of MMP-7 and c-Met. However, miR inhibition of Ets-1 only partially replicated the effects of truncated syndecan-1. Because reporter assays in syndecan-1 transfectants revealed decreased promoter binding of AP-1 as well as Ets-1, Ets-1 silencing alone may not be sufficient to mimic all effects of syndecan-1 overexpression. Since the truncated form of syndecan-1 proved to be more effective, we speculate that post shedding stub of syndecan-1 may regulate cell differentiation and proliferation. Given the plethora of functions of syndecan-1 in the liver, additional beneficial effects of syndecan-1 re-expression in HCC can be expected.

## Supplementary Materials

The following are available online at https://www.mdpi.com/2218-273X/10/10/1356/s1, Figure S1: Expression of c-Met in HepG2 and Hep3B cells, Figure S2: Zymography of HepG2 and Hep3B conditioned cell media, Figure S3: PMA treated HepG2 conditioned cell media, Table S1: qRT-PCR primer and oligo sequences, Table S2: Antibodies used.
