# Supplementary material for: Syndecan-1 Promotes Hepatocyte-Like Differentiation of Hepatoma Cells Targeting Ets-1 and AP-1
Source: Biomolecules. 2020 Sep 23;10(10):1356. doi: 10.3390/biom10101356 (PMC7598270; doi:10.3390/biom10101356)
Supplement: Supplementary file 1 [file biomolecules-10-01356-s001.pdf]

biomolecules

Article

Syndecan-1 promotes hepatocyte-like differentiation of hepatoma cells targeting Ets-1 and AP-1

Péter Hollósi [1,2†], Lóránd Váncza [1†], Katalin Karászi [1†], Katalin Dobos [1], Bálint Péterfia [1,3], Enikő Tátrai [1,4], Péter Tátrai [1,5], Tibor Szarvas [1,6,7], Sándor Paku [1], László Szilák [8] and Ilona Kovalszky [1,*]

[1] First Department of Pathology and Experimental Cancer Research, Semmelweis University, Üllői út 26, H-1085 Budapest, Hungary; phollosi@yahoo.com (P.H.); vancza_lorand@yahoo.com (L.V.); tika0604@gmail.com (K.K.); kdobos@gmail.com (K.D.); peterfiab@gmail.com (P.B.); tatraien@gmail.com (E.T.); soobaab@gmail.com (P.T.); sztibusz@gmail.com (T.S.); paku.sandor@med.semmelweis-univ.hu (S.P.); kovalszky.ilona@med.semmelweis-univ.hu (I.K.)
[2] Tumor Progression Research Group of Joint Research Organization of Hungarian Academy of Sciences and Semmelweis University, Széchenyi István tér 9, H-1051 Budapest, Hungary;
[3] Faculty of Information Technology and Bionics, Pázmány Péter Catholic University, Práter u. 50/A, H-1083 Budapest, Hungary;
[4] Department of Experimental Pharmacology, National Institute of Oncology, Ráth György utca 7-9, H-1122 Budapest, Hungary;
[5] Solvo Biotechnology, Irinyi József utca 4-20, H- 1117 Budapest, Hungary;
[6] Department of Urology, Semmelweis University, Üllői út 78/B, H-1082 Budapest, Hungary;
[7] Department of Urology, West German Cancer Center, University of Duisburg-Essen, University Hospital Essen, Hufelandstr. 55, 45147, Essen, Germany;
[8] Szilak Laboratories Bioinformatics and Molecule-Design, Gem utca 14, H-6723 Szeged, Hungary; laszlo.szilak@gmail.com (L.S.)
* Correspondence: kovalszky.ilona@med.semmelweis-univ.hu; Tel.: +36-1-459-1500 (ext. 54449)
† These authors contributed equally to this paper.

Supplementary information.

Contents

Table S1. qRT-PCR primer and oligo sequences.
Table S2. Antibodies used.
Figure S1. Expression of c-Met in HepG2 and Hep3B cells.
Figure S2. Zymography of HepG2 and Hep3B conditioned cell media.
Figure S3. PMA treated HepG2 conditioned cell media.

Table S1. qRT-PCR primer and oligo sequences.

Gene	Primer /oligo	Sequence (5'–3')	Amplicon length (bp)
MMP7	F	GTGGTCACCTACAGGATCGTA	492
	R	CTGAAGTTTCTATTTCTTTCTTGA	
ETS1	F	TTCACTAAAGAACAGCAAC	205
	R	TGTCCCCAACAAAGTCTG	
Syndecan-1 ED	F	GCTGACCTTCACACTCCCCA	124
	R	CAAAGGTGAAGTCCTGCTCCC	
Syndecan-1 CD	F	GAAGAAGGACGAAGGCAG	83
	R	CCTCCTGTTTGGTGGGC	
miR-lacZ	S	TGCTGAAATCGCTGATTTGTGTAGTCGTTTTGGC	-
		CACTGACTGACGACTACACATCAGCGATTT	
	A	CCTGAAATCGCTGATGTGTAGTCGTCAGTCAGT	
		GGCCAAAACGACTACACAAATCAGCGATTTC	
miR-362	S	TGCTGAATAGTGGACATCTGCACATGTTTTGGC	-
		CACTGACTGACATGTGCAGGTCCCACTATTG	
	A	CCTGAATAGTGGGACCTGCACATGTCAGTCAGT	
		GGCCAAAACATGTGCAGATGTCCCACTATTC	
miR-641	S	TGCTGTCTAGATGTTCCCATAAGATGGTTTTGG	-
		CCACTGACTGACCATCTTATGAACATCTAGA	
	A	CCTGTCTAGATGTTCATAAGATGGTCAGTCAGT	
		GGCCAAAACCATCTTATGGGAACATCTAGAC	

Abbreviations: F: forward primer, R: reverse primer, S: sense oligo, A: antisense oligo, bp: base pair; ED: ectodomain, CD: cytoplasmic domain

Table S2. Antibodies used.

Primary antibody	Host species, isotype	Manufacturer*	Cat. No.	Dilution IF#	Dilution blot#
p38 MAPK	rabbit polyclonal IgG	Cell Signaling Technology	9212	-	1:500
phospho-p38 MAPK (Thr180/Tyr182) XP®	rabbit monoclonal IgG, clone D3F9	Cell Signaling Technology	4511	-	1:500
p44/42 MAPK (Erk1/2)	rabbit polyclonal IgG	Cell Signaling Technology	9102	-	1:500
phospho-p44/42 MAPK (Erk1/2) (Thr202/Tyr204) XP®	rabbit monoclonal IgG, clone D13.14.4E	Cell Signaling Technology	4370	-	1:500
Syndecan-1 (ectodomain)	mouse monoclonal IgG, clone B-B4	AbD Serotec	MCA681	1:100	-
Ets-1 (C-20)	rabbit polyclonal IgG	Santa Cruz Biotechnology	sc-350	1:100	1:500
Desmoplakin	rabbit polyclonal IgG	Abcam	ab14418	1:100	-
Albumin	rabbit polyclonal IgG	Dako	a0001	1:100	-
Heparan sulfate (HS4C3)	Phage display antibody containing a VSV-G tag	kind gift from TH van Kuppevelt	-	1:2	-
Heparan sulfate (AO4B08)	Phage display antibody containing a VSV-G tag	kind gift from TH van Kuppevelt	-	1:2	-
Vesicular stomatitis virus glycoprotein (VSV-G)	mouse monoclonal IgG, clone P5D4	Sigma-Aldrich Co.	SAB4200 695	1:100	-
MMP-7	rabbit polyclonal IgG	Sigma-Aldrich Co.	AV46075	1:100	-
Met (c-28)	rabbit polyclonal IgG	Santa Cruz Biotechnology	sc-161	-	1:500
**Secondary antibody**	**Host species, isotype**	**Manufacturer***	**Cat. No.**	**Dilution IF#**	**Dilution blot#**
Anti-rabbit Ig/HRP	Goat polyclonal	Dako Cytomation	P0448	-	1:2,000
Alexa Fluor® 568 anti-rabbit IgG (H+L)	Goat polyclonal	Invitrogen	A11011	1:200	-
Alexa Fluor® 568 anti-mouse IgG (H+L)	Goat polyclonal	Invitrogen	A11004	1:200	-
Alexa Fluor® 488 anti-rabbit IgG (H+L)	Goat polyclonal	Invitrogen	A11008	1:200	-

* Cell Signaling Technology, Danvers, MA, USA; AbD Serotec Kidlington, UK; Santa Cruz Biotechnology, Inc Dallas, TX, USA; Abcam, Cambridge, UK; Dako/DakoCytomation, Agilent Technologies, Inc., Santa Clara, CA, USA; Invitrogen by Life Technologies, Carlsbad, CA, USA; Sigma-Aldrich Co., St. Louis, MO, USA; kind gift from TH van Kuppevelt; Nijmegen, The Netherlands

#**IF**: immunofluorescent cytochemistry, **blot**: Western blot or dot blot

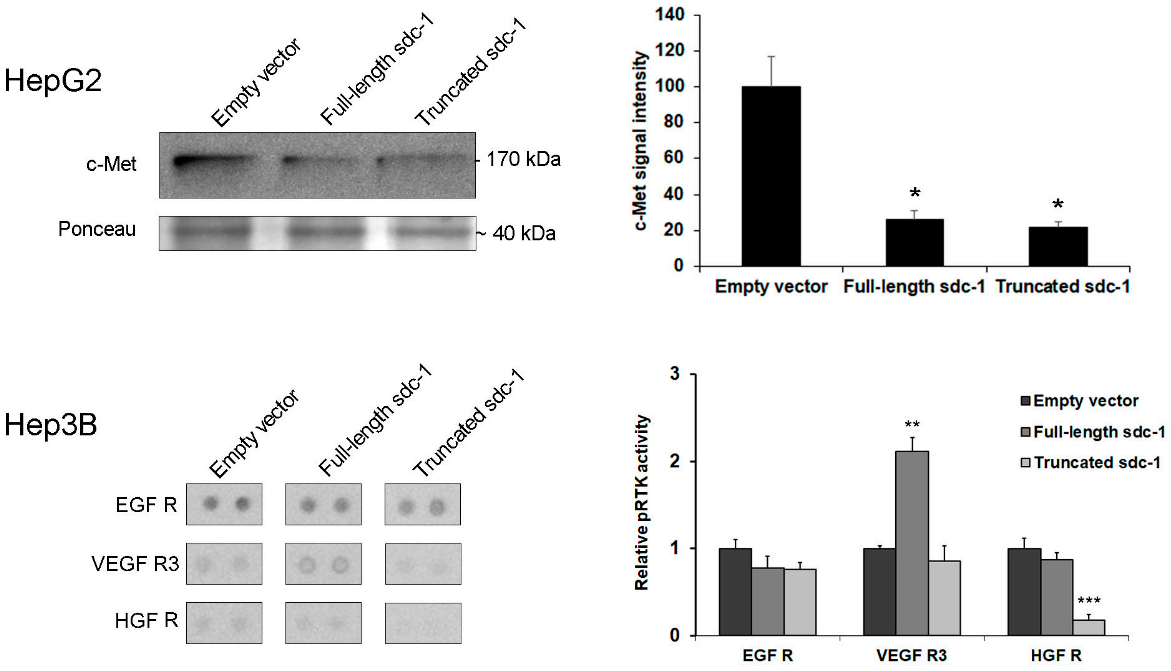

Figure S1. Expression of c-Met in HepG2 and Hep3B cells. Western blot of c-Met of HepG2 and pRTK array of Hep3B showing expression of EGFR, VEGFR3 and HGFR. Data points represent the mean ± SD, n = 2; * $p < 0.05$; ** $p < 0.01$; *** $p < 0.001$ versus empty vector transfectant.

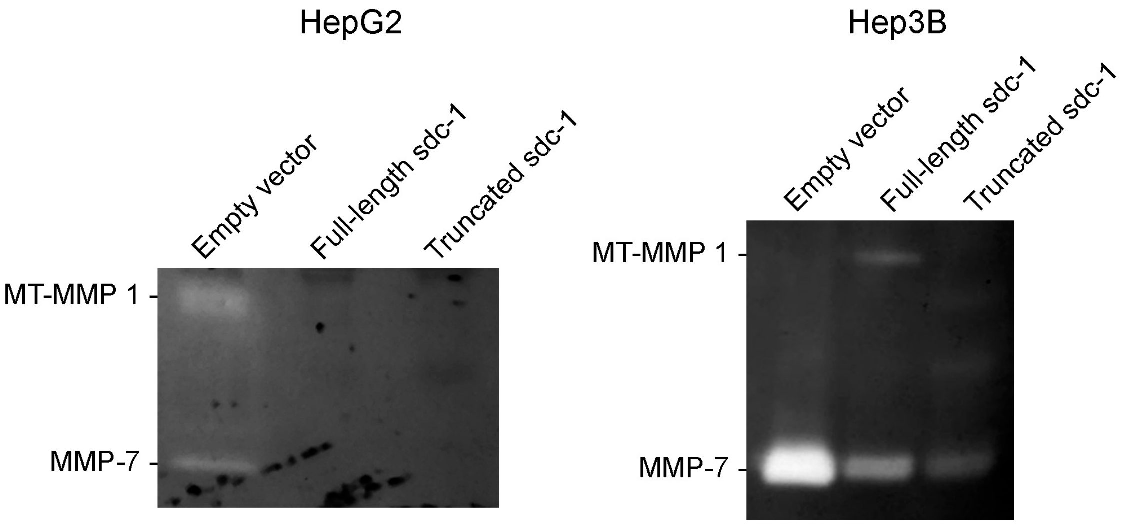

Figure S2. Zymography of HepG2 and Hep3B conditioned cell media.

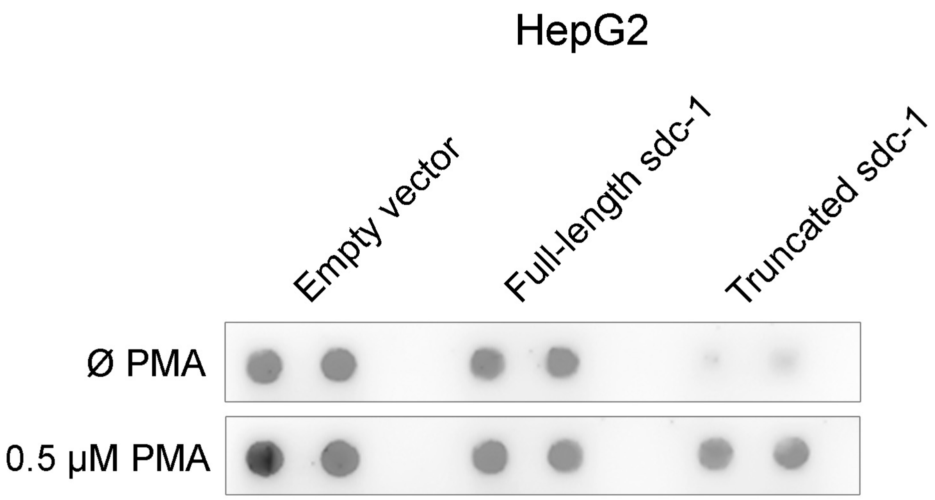

Figure S3. PMA treated HepG2 conditioned cell media.
